# Variation in phylogenetic tendencies of contiguous riboswitches

**DOI:** 10.1099/mgen.0.001496

**Published:** 2025-09-17

**Authors:** Nataly Morales, Enrique Merino

**Affiliations:** 1 Department of Molecular Microbiology, Instituto de Biotecnología, Universidad Nacional Autónoma de México, Cuernavaca, Morelos CP 62210, Mexico

**Keywords:** Cluster of Orthologous Group (COG), contiguous riboswitches, phylogenetic group, regulatory domain, sensor domain

## Abstract

Riboswitches are widely distributed RNA regulatory elements primarily found in prokaryotes. Over a decade ago, it was discovered that certain genes are regulated either by *multiple tandemly arranged riboswitches* or by *riboswitches with tandem sensor domains*.

Riboswitches consist of a sensor domain, also known as an aptamer, and an expression platform, primarily regulating gene expression through transcription or translation attenuation, although other mechanisms such as RNA splicing or stability modulation are also possible. In *tandemly arranged riboswitches*, the first riboswitch is often positioned far from the ribosome binding site of the target gene, making transcriptional regulation a likely primary mechanism. In contrast, *riboswitches with adjacent aptamers* have a single expression platform, which may be located at varying distances from the regulated gene. Consequently, no inherent bias towards either transcriptional or translational regulation is expected in this case.

Considering the reported enrichment of transcriptional riboswitches in certain phylogenetic clades, the present study analyses the distribution of *tandemly arranged riboswitches* and *riboswitches with adjacent aptamers* across various phylogenetic groups, as well as the tendencies of different riboswitch families to regulate genes in a contiguous fashion. Our results indicate that *Bacillota* is the phylum with the most notable families of *tandemly arranged riboswitches*. In contrast, regulation of gene expression via *tandem sensor domains* was predominantly associated with the *Pseudomonadota* phylum. On the other hand, our statistical analysis identified the T-box and cyclic dimeric guanosine monophosphate as the most prominent members of *tandemly arranged riboswitches*, while the glycine riboswitch is the most relevant family to be composed of *adjacent aptamers*.

In addition, this article presents a comprehensive analysis of all relevant orthologous gene groups regulated by contiguous riboswitches, highlighting trends in cases where strict regulation is necessary due to the high metabolic cost of a pathway. Finally, we provide an in-depth discussion of the observed regulatory tendencies.

## Data Summary

Genomes are accessible in Kyoto Encyclopedia of Genes and Genomes, KEGG database 2022 (https://www.genome.jp/kegg/genome/), and covariance models describing riboswitches are in the Rfam database v.14.0 (https://ftp.ebi.ac.uk/pub/databases/Rfam/). We used the motif search algorithm of the Infernal (Inference of RNA alignments) package, the *Cmsearch* program.

**Impact Statement**

Here, through *in silico* identification and comprehensive statistical analysis of all known riboswitch families across complete, non-redundant genome sequences, we uncovered the key cellular processes regulated by contiguously arranged riboswitches. Additionally, we found that tandemly arranged riboswitches, which utilize Rho-independent transcriptional terminators as expression platforms for the first riboswitch, are more prevalent in *Bacillota*. In contrast, tandem sensor domains without intervening regulatory platforms between aptamer domains exhibit a notable enrichment in *Pseudomonadota*.

## Introduction

Riboswitches are RNA regulators located within intergenic regions in certain messenger RNA molecules of bacteria, archaebacteria and some eukaryotes. They possess the ability to switch between turning on and turning off genetic expression without the need for any protein participation [1,5]. Commonly, riboswitches consist of two structural domains: an aptamer, or sensor domain that adopts a tridimensional structure involved in recognizing a specific target molecule with high affinity and great specificity, and an expression platform, or regulatory domain that directly controls the expression of genes located downstream by the formation of secondary RNA structures that includes transcription and translation attenuators or ribozymes. These structures are determined by allosteric changes induced in the sensor after the union of its corresponding ligand. In addition, some riboswitches might act in trans, like small RNAs [4,6]. Riboswitches regulate a variety of genes codifying proteins related to biosynthesis and transport of metabolites, such as carbohydrates (glucosamine-6-phosphate), coenzymes (flavin mononucleotide, thiamine pyrophosphate, cobalamin, tetrahydrofolate), nucleotides [adenine, guanine, cyclic dimeric guanosine monophosphate (c-di-GMP), pre-queuosine, ATP], amino acids (lysine, glycine, SAM, *S*-adenosyl homocysteine), ions (molybdenum, magnesium), second messengers (cyclic AMP, cyclic GMP) and vitamins (cobalamin) [1,5].

The conservation of sequence and structure of riboswitches is a characteristic present in riboswitches that results from the high selectivity of the aptamer for its corresponding ligand. Exploiting this conservation, computational analyses based on covariance models have been carried out [2,5, 7]. The main database containing covariance models describing riboswitches is Rfam [8]. In our study, we used Rfam version 14.0, which includes 50 models describing riboswitches. Thorough analyses of these regulatory elements across the genomes of representative organisms have revealed that certain classes of riboswitches are broadly distributed [flavin mononucleotide (FMN);, thiamine pyrophosphate (TPP) and *S*-adenosylmethionine (SAM)], while some others are restricted to specific phylogenetic clades (for example, T-box in *Bacillota*, fluoride in *Methanobacteriota* and glutamine in *Pseudomonadota*) [5,7, 9].

Commonly, only one copy of a riboswitch is present in the 5′ upstream region of the regulated gene. However, in certain instances, multiple *tandemly arranged riboswitches* or *riboswitches with adjacent aptamers* have been documented. The term ‘tandem riboswitches’ has been used to define these kinds of riboswitches [10,13]. Currently, only contiguous riboswitches of classes Gly, TPP, SAM, c-di-GMP, cobalamin, purine and guanidine have been experimentally characterized [14]. In addition, T-boxes and other *tandemly arranged riboswitches* were identified by computational analyses [5,15]. Moreover, the regulatory outcomes of tandem riboswitches might be complex and depend on their different architectures, and their regulatory behaviours have recently been described as logic gates, whose behaviours depend fundamentally on the following properties: (A) whether their aptamer domains sense the same or distinct ligand-binding molecules; (B) whether they consist of two independent riboswitch units or two adjacent aptamers followed by a single regulatory platform; and (C) whether the regulatory platforms of the contiguous riboswitches are of the same type, transcriptional or translational, or of different types [16].

**Fig. 1.**
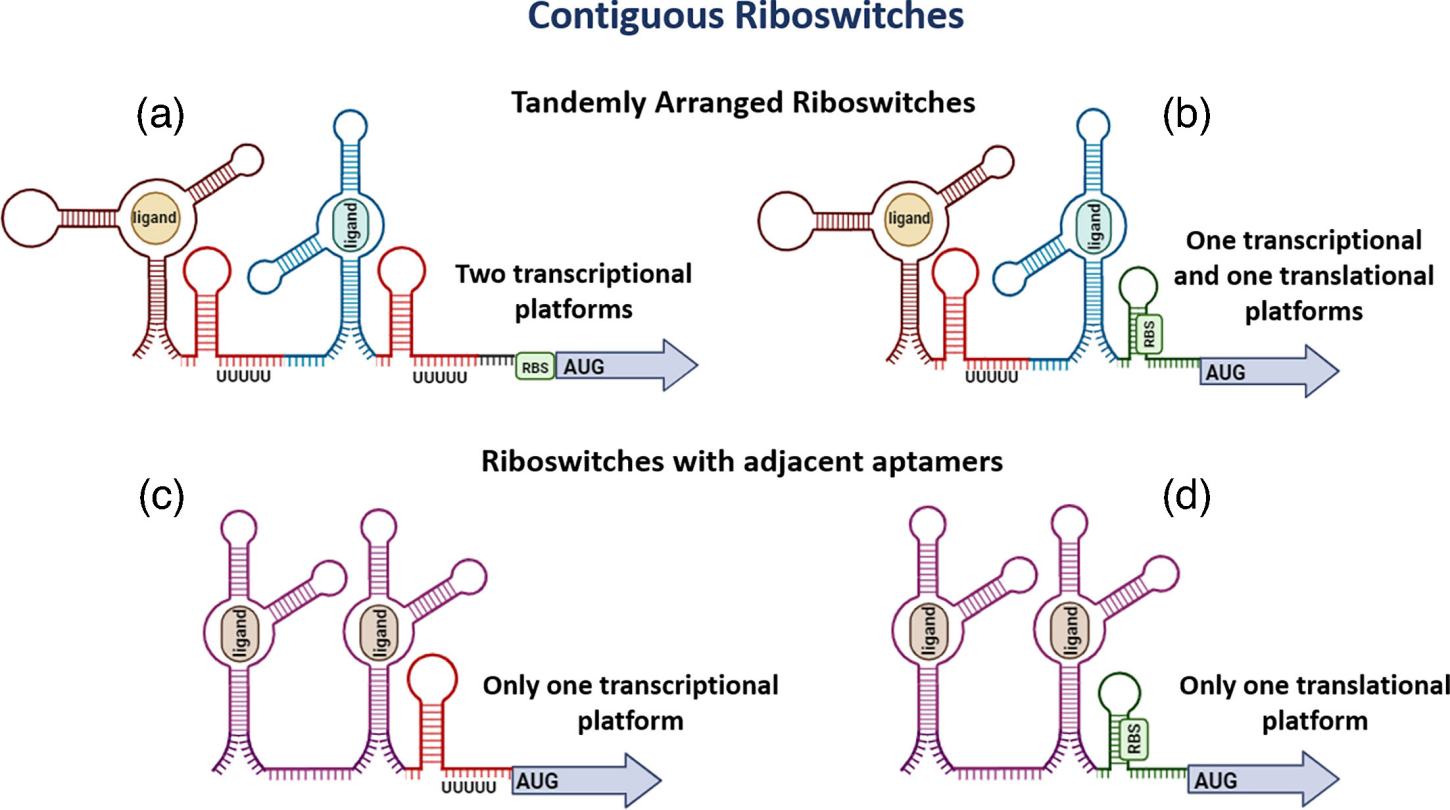
Schematic representation of contiguous riboswitches. (**a**–d) Contiguous riboswitches controlling a gene or an operon are in the same intergenic region. They can be formed by two, three or more aptamers, which can sense the same (homogeneous, represented by structures of the same colour) or different (heterogeneous, represented by structures of different colours) types of ligands. (**a**, b) show *tandemly arranged riboswitches*, each composed of one aptamer and one expression platform. (**c**, d) show *riboswitches with adjacent aptamers*, where the set of riboswitches has two or more sensor domains followed by a single expression platform.

 The working hypothesis of our study considers that, in *tandemly arranged riboswitches*, the first riboswitch predominantly regulates transcription, while the second riboswitch is not constrained in terms of regulatory level. This is because the first riboswitch is typically positioned far from the ribosome-binding site, preventing interaction, whereas the second riboswitch has no such positional restriction. As a result, the second riboswitch may regulate either transcription or translation, allowing greater diversity in regulatory mechanisms (Fig. 1a, d). Conversely, for riboswitches composed of two or more *adjacent aptamer domains* followed by a single expression platform, no bias toward a specific regulatory mechanism is expected, as the expression platform’s distance from the regulated gene varies (Fig. 1c, d). An example of this type of riboswitch is the glycine riboswitch.

These regulatory elements were predicted from computer analysis of genome sequences from the Kyoto Encyclopedia of Genes and Genomes (KEGG) database. A statistical analysis of the use of the different kinds of riboswitches’ regulatory platforms has shown a significant bias for transcriptional regulatory platforms in *Bacillota* and *Mycoplasmatota*. In contrast, a tendency to use translational riboswitches has been found in *Pseudomonadota* and *Actinomycetota* [17].

Considering the information provided on the phylogenetic tendencies in the use of transcriptional versus translational platforms, this study aims to examine potential biases in the distribution of different tandem riboswitch architectures among prokaryotic groups. Our findings reveal that *Bacillota* contains the highest number of *riboswitches arranged in tandem*, while *riboswitches with adjacent aptamers* are significantly enriched in *Pseudomonadota*.

Finally, our work includes a comprehensive analysis of the prevalence of regulation mediated by contiguous riboswitches in specific genes and biochemical processes, along with a discussion of their potential implications.

## Methods

### Sequence data

Genome sequences of non-redundant prokaryotic organisms were retrieved from the KEGG database 2022 [18] (https://www.genome.jp/kegg/genome/). This database contained 6,889 bacterial and 380 archaeal circular curated genomes. From this set of sequences, we selected the species with the largest number of genes among each species’ strains and ended with a set of 5,418 genome sequences of representative organisms distributed in 37 distinct phylogenetic clades (Table S1, available in the online Supplementary Material).

### Clustering of riboswitches into families in accordance with their binding molecules

Considering the nature of their ligands, each one of the 50 classes of riboswitch aptamers described in the Rfam database version 14.0 [8], we clustered them into 27 families. The families with more members correspond to SAM (SAM, SAM-IV, SAH, SAM_alpha, SAM-I-IV, SAM-III, SAM-SAH, SAM_V and SAM_VI) and c-di-GMPc (c-di-GMP-I, c-di-GMP-II, c-di-GMP-I-GGC, di-GMP-II-GAG, c-di-GMP-I-UAU and c-di-GMP-II-GCG), while other families contained only one member, such as T-box, TPP and FMN [19] (Table S2).

### Identification of contiguous riboswitches

Thanks to the structure and sequence conservation of aptamer domains of riboswitches, we localized these elements on untranslated regions of genomes from chosen bacteria and archaea. We used the covariance models of riboswitch aptamers that have been reported in Rfam version 14.0 [8] and the motif search algorithm of the Infernal (Inference of RNA alignments) package, Cmsearch program [20].

Although Rfam provides three standard bit score thresholds for each covariance model, GA (gathering), noise cutoff and trusted cutoff, we observed that in certain cases, such as the T-box riboswitch aptamer, these thresholds were too stringent. As a result, several *bona fide* riboswitch aptamers were excluded, despite being located upstream of genes typically regulated by these elements (e.g. aminoacyl-tRNA synthetases, amino acid biosynthesis or transport genes).

To address this, we developed an analytical procedure to empirically refine the bit score cutoff values for each riboswitch aptamer family. For every intergenic region upstream of each gene across the 5,418 genomes included in our dataset, we generated 100 random sequences of the same length, preserving the dinucleotide composition of the corresponding genome. This yielded a total of 1,996,145,900 randomized intergenic sequences.

Using Cmsearch, we scanned the set of random sequences with each of the 50 riboswitch covariance models to determine the highest bit score obtained from non-specific (random) hits. These values were then used to establish revised cutoff thresholds aimed at discriminating *bona fide* riboswitch aptamers from spurious matches. As shown in Table S3, most riboswitch families exhibited similar bit score thresholds, with a few exceptions, such as the glycine-GGAnGA (RF03166), raiA RNA (RF03072) and T-box (RF00230) models.

We applied these refined bit score thresholds to define riboswitch aptamer candidates in our study. Table S4 presents representative examples of riboswitches identified using our adjusted cutoffs that would have been excluded under the original Rfam GA thresholds. Notably, these candidates are consistent with previously described riboswitch-mediated regulation in orthologous genes, providing strong evidence to support their inclusion in our analysis. Then, we classified a pair of riboswitches as *contiguous* if they were in the same intergenic region.

### Identification of Rho-independent transcription terminators in the regulatory domain of riboswitches

We employed a previously developed programme within our research group to identify potential Rho-independent transcription terminators in the regulatory domains of riboswitches [7]. In brief, this method utilizes a 50-nt analysis window downstream of each region identified as a riboswitch. Within this analytical window, we searched for a run of at least nt containing a minimum of 5 T residues. Subsequently, employing the RNAfold program version 2.4.0 [21], we look for the most stable RNA secondary structure that formed a stem and loop structure with a Gibbs free energy of less than −10 kcal mol^−1^. In cases where the analysed region could form multiple stem and loop structures, we considered only the one nearest to the run of T residues. We allowed for 2 nt between the base of the secondary structure and the run of T residues.

### Classification of contiguous riboswitches

We consider a set of riboswitches as *contiguous riboswitches* if they are in a common intergenic region and regulate the same gene or set of genes. In addition, we classify contiguous in two different categories: *tandemly arranged riboswitches* and *riboswitches with adjacent aptamers*.

We defined *tandemly arranged riboswitches* as contiguous riboswitches consisting of at least two units, each with its own aptamer and regulatory domains. Typically, the regulatory domain of the first riboswitch features a Rho-independent transcription terminator; however, there is no clear trend regarding the regulatory domain of the second riboswitch. (Fig. 1a, b).

Conversely, we defined *riboswitches with adjacent aptamers* as those consisting of at least two adjacent recognition domains followed by a single regulatory platform. In such instances, the regulatory domain is situated immediately after the second aptamer (Fig. 1c, d). Given that the typical size of Rho-independent transcription terminators is more than 20 nt (as shown in Fig. S1), we classified riboswitches as having tandem adjacent aptamer domains when the distance between their aptamer domains was fewer than this specified number of nucleotides.

Additionally, we classified the *contiguous riboswitches* based on the homogeneity of the molecules recognized by their aptamers: homogeneous if they recognize the same molecule and heterogeneous otherwise.

### Enrichment analysis of riboswitches

Table 1 lists the type of enrichment analyses performed in our study and their corresponding variables. All the enrichments were evaluated using the hypergeometric probability implemented in the *phyper* and *dhyper* functions of the RStudio software version 4.2.1.

**Table 1.** Types of enrichment analyses and corresponding variables

Type of enrichment	Entire population		Group under analysis	
	No. of element	No. of success	No. of element	No. of success
S6. Enrichment of a phylum to have riboswitches	Number of genes	Number of riboswitches	Number of genes by phylum	Number of riboswitches by phylum
S7. Enrichment of a phylum to have contiguous riboswitches	Number of genes	Number of contiguous riboswitches	Number of genes by phylum	Number of contiguous riboswitches by phylum
S9. Enrichment of a phylum to have tandemly arranged riboswitches	Number of genes	Number of tandemly arranged riboswitches	Number of genes by phylum	Number of tandemly arranged riboswitches by phylum
S10. Enrichment of a phylum to have tandemly arranged aptamers	Number of genes	Number of adjacent aptamers	Number of genes by phylum	Number of adjacent aptamers by phylum
S11. Enrichment of riboswitch families arranged contiguously	Number of riboswitches	Number of contiguous riboswitches	Number of riboswitches by family	Number of contiguous riboswitches by family
S12. Enrichment of riboswitch families with tandem riboswitches	Number of riboswitches	Number of contiguous riboswitches with tandemly arranged riboswitches	Number of riboswitches by family	Number of tandem riboswitches per family among contiguous riboswitches
S13. Enrichment of riboswitch families with tandem aptamers	Number of riboswitches	Number of contiguous riboswitches with adjacent aptamers	Number of riboswitches by family	Number of contiguous riboswitches with adjacent aptamers per family
S14. Enrichment of riboswitch families within specific bacterial phyla	Number of riboswitches	Number of riboswitches by family	Number of riboswitches belonging to a phylum	Number of riboswitches by family belonging to a phylum
S15. Enrichment of contiguous riboswitch families by phylum	Number of riboswitches	Number of riboswitches by family	Number of riboswitches belonging to a phylum	Number of contiguous riboswitches by family belonging to a phylum
S16. Enrichment of a COG to be regulated by riboswitches	Number of genes	Number of genes regulated by riboswitches	Number of genes by COG	Number of genes by COG regulated by riboswitches
S17. Enrichment of a COG to be regulated by riboswitches in a phylum	Number of genes by COG	Number of genes by COG regulated by riboswitches	Number of genes by COG by phylum	Number of genes by COG by phylum regulated by riboswitches
S18. Enrichment of a COG to be regulated by contiguous riboswitches	Number of genes	Number of genes regulated by contiguous riboswitches	Number of genes by COG	Number of genes by COG regulated by contiguous riboswitches
S19. Enrichment of a COG to be regulated by contiguous riboswitches in a phylum	Number of genes by COG	Number of genes by COG regulated by contiguous riboswitches	Number of genes by COG by phylum	Number of genes by COG by phylum regulated by contiguous riboswitches

### Clustering of genes based on their orthologous relationships

Genes were clustered into their respective orthologous groups based on the Cluster of Orthologous Groups (COG) database [22]. For genes without a COG assignment, we utilized our previous classification of orthologous groups, which was established through a clustering procedure based on bi-directional best hits among our set of fully sequenced genomes [23].

## Results and discussion

In this current study, we investigate the occurrence of *contiguous riboswitches* within a diverse selection of representative prokaryotic organisms. We aim to identify discernible patterns or biases in the distribution of tandem riboswitches, investigating whether they are related to phylogeny, the riboswitch family or the type of expression platform employed to regulate gene expression.

Furthermore, we describe the statistical enrichment of specific families of orthologous genes, identified by their corresponding COG groups, which are regulated by *contiguous riboswitches*, and we propose potential biochemical explanations for this distinctive mode of regulation.

### Differential riboswitch prevalence across phylogenetic clades

**Fig. 2.**
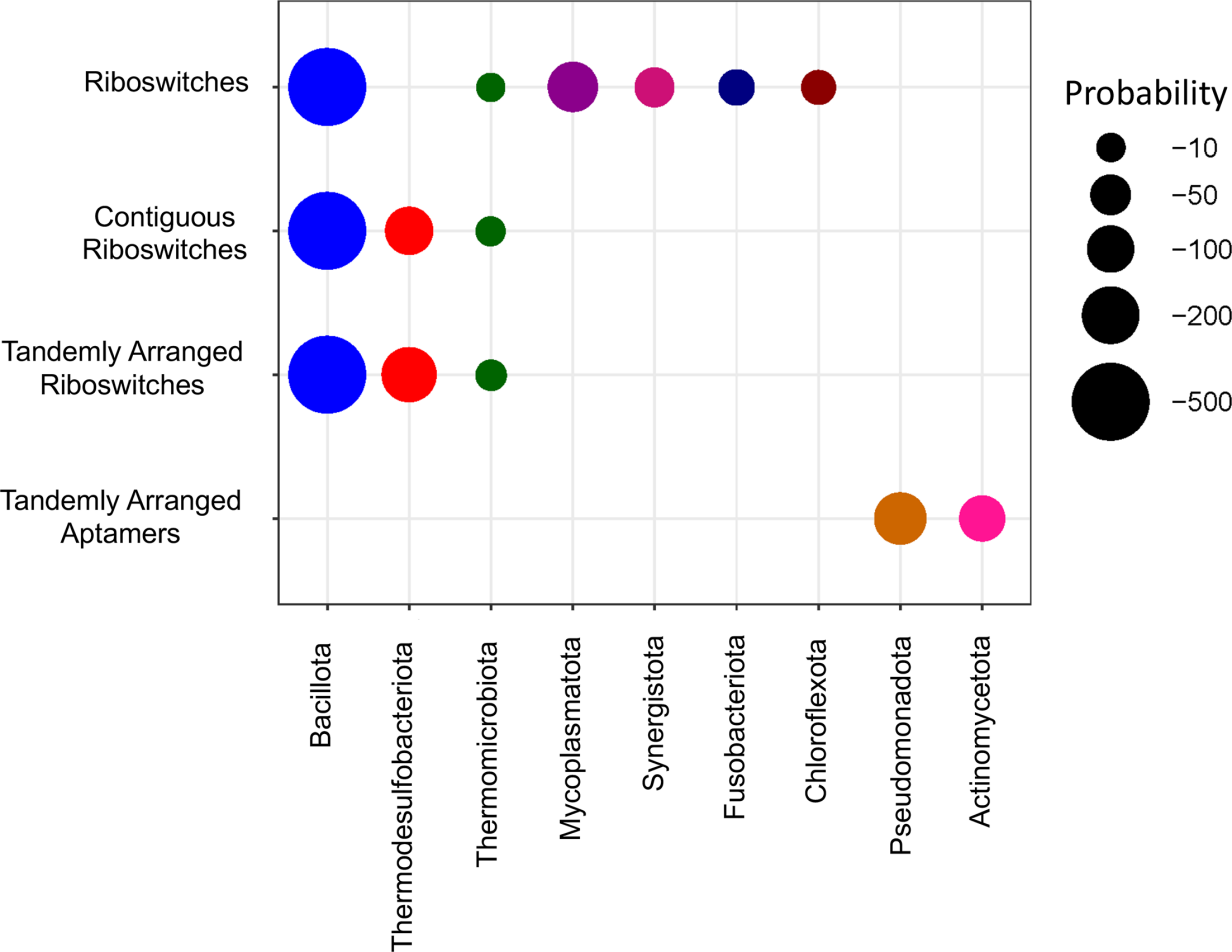
Enrichment of riboswitch configurations across bacterial phyla. The size of the circles represents the *P*-values obtained from our statistical analysis using the hypergeometric distribution (see the ‘Methods’ section). In this context, the *P*-value indicates the probability of observing the same or a greater number of occurrences of a specific riboswitch configuration within a given phylum, based on its overall frequency across all organisms included in the study. The riboswitch configurations considered in this analysis were (i) riboswitches, encompassing all riboswitch families in any structural organization; (ii) contiguous riboswitches, two or more riboswitches located within the same intergenic region; (iii) tandemly arranged riboswitches, contiguous riboswitches, each composed of an independent sensing domain and regulatory platform; (iv) tandemly arranged aptamers, configurations consisting of two or more sensing domains (aptamers) followed by a single shared regulatory domain located downstream of the final aptamer. The input variables used in the hypergeometric test for this analysis are detailed in Table 1. Circle colours indicate the different phyla analysed in our study.

As a starting point for our study, we investigated whether the occurrence of riboswitches varies in frequency among different phylogenetic clades. Using 50 riboswitch covariance models from the Rfam database version 14.0 [8], we searched for riboswitch aptamers in the intergenic regions of our set of 5,418 non-redundant genome sequences. We identified a total of 70,892 riboswitches, which were further clustered into 27 groups based on the similarities of their recognition molecules (Tables S2, S5). To assess this, we evaluated the statistical significance of riboswitch enrichment in different phylogenetic clades using the hypergeometric distribution (see the ‘Methods’ section). The statistical values of the most significant enrichments are presented in the first line of Fig. 2, labelled ‘Riboswitches’, and Table S6. For a complete reference of the variable involved in this enrichment analysis, see Table 1.

From Fig. 2 and Table S6, *Bacillota* stands out as the phylum with the most significant statistical enrichment of riboswitches, followed by a less significant yet notable enrichment in *Mycoplasmatota*, *Synergistota*, *Fusobacteriota* and *Chloroflexota*.

*Pseudomonadota* merit special mention. Although riboswitch regulation has been well-documented in this phylum [24], the number of riboswitches identified in our in silico study in this phylum, 17,605 out of 8,493,198 genes (0.21%), is not statistically significant when compared to the total number of riboswitches (70,892) found in the 19,940,848 genes across all 5,418 representative organisms in our study (0.36%) (see Table S6).

### Distribution of contiguous riboswitches across phylogenetic clades

Most riboswitches typically occur as a single copy to regulate the expression of their target genes. However, certain riboswitch families occasionally appear in multiple, contiguous copies, working together to exert their regulatory responses. In a second analysis, we identified riboswitches that appeared in multiple copies within the same regulatory region. In our study, these riboswitches correspond to the class of contiguous riboswitches, with a total count of 2,944. To determine potential phylogenetic tendencies for the presence of these contiguously arranged riboswitches, we performed a hypergeometric distribution analysis for each prokaryotic phylum. The values of the most prominent enrichments are presented in the second line of Fig. 2, labelled ‘Contiguous riboswitches’, and Table S7. For a comprehensive reference of all the phyla and variables involved in this enrichment analysis, see Table 1.

Our results on the enrichment of contiguous riboswitches clearly align with the enrichment observed in the number of riboswitches per phylum (Table S7, fifth and sixth columns). *Bacillota* stands out as the phylogenetic phylum with the most significant enrichment of contiguous riboswitches.

### Types and classification of contiguous riboswitches

To further characterize the enrichment tendencies of contiguously arranged riboswitches in specific phylogenetic clades, we classified them into two categories: *tandemly arranged riboswitches* and *riboswitches with adjacent aptamers*.

*Tandemly arranged riboswitches* consist of at least two riboswitch units, each containing one aptamer and one expression platform (as shown in Fig. 1a, b), whereas *riboswitches with adjacent aptamers* contain two or more aptamers followed by a single expression platform after the final aptamer in the set (illustrated in Fig. 1c, d). To differentiate between these two types of riboswitch arrangements, we searched for Rho-independent transcription terminators after the first aptamer in pairs of contiguous riboswitches (see ‘Methods’ section for details). Among our collection of 1,442 inter-aptameric sequences, we successfully identified 805 sequences with at least 1 region whose transcribed secondary structure resembles a Rho-independent transcription terminator. The sequences and folding patterns of these terminators are presented in Table S8. Riboswitch pairs with a Rho-independent transcription terminator immediately after the first aptamer were classified as *tandemly arranged riboswitches*.

To further distinguish *tandemly arranged riboswitches* from *riboswitches with adjacent aptamers*, we plotted and compared the distances between the aptamers of contiguous riboswitches in cases where a Rho-independent transcription terminator was identified versus those where this regulatory element was not found. From this analysis, we concluded that a minimum of 20 nt between the aptamers of *tandemly arranged riboswitches* is commonly needed to encompass a Rho-independent transcription terminator (see Fig. S1 and the ‘Methods’ section). Considering this distance, riboswitches with aptamer domains that were closer than 20 nt were considered as *riboswitches with adjacent aptamers*.

### Phylogenetic enrichment of tandemly *arranged riboswitches*

Following the identification of *tandemly arranged riboswitches* in our set of genome sequences, we used the hypergeometric distribution to evaluate their enrichment across different phylogenetic clades. Notably, *Bacillota* exhibited a highly significant enrichment (see Fig. 2, third line, and Table S9). This result aligns with three main ideas. First, as shown in our previous analyses, *Bacillota* is the phylum with the most significant enrichment of riboswitches, whether considered as individual elements (see Fig. 2, first line) or when these riboswitches are found contiguously regulating the same gene (see Fig. 2, second line). Second, our working hypothesis states that in a pair of contiguous riboswitches arranged side by side, the first riboswitch often lacks proximity to the ribosome binding site of the target gene; consequently, the first riboswitch tends to regulate transcriptionally. Third, previous analyses by our group have identified *Bacillota* as the phylogenetic clade with the highest number of riboswitches containing transcriptional regulatory platforms [17]. Taking these arguments together, it is consistent with our results that *Bacillota* show a more significant tendency to present *tandemly arranged riboswitches* compared to other phylogenetic clades. In addition to *Bacillota*, marginal enrichments of *tandemly arranged riboswitches* are observed in the *Thermodesulfobacteriota* and *Thermomicrobiota* phyla (Fig. 2, third line and Table S9).

The regulatory outcomes of *tandemly arranged riboswitches* that function independently have been previously described [13,16]. Firstly, *tandemly arranged riboswitches* that sense identical ligands are expected to exhibit pseudo-cooperative responses. Secondly, in the case of *tandemly arranged riboswitches* sensing different ligands, more complex outputs are anticipated. These regulatory outputs have been theoretically described as two-input Boolean logic gate systems [10,16]. In addition, the outcomes of *tandemly arranged riboswitches* may also depend on whether their regulation occurs at the same or different levels, transcriptionally or translationally. For *tandemly arranged riboswitches* with regulatory platforms functioning at the same level, either both transcriptionally or both translationally, a pseudo-cooperative dose-response is expected [16]. Conversely, for *tandemly arranged riboswitches* with regulatory platforms operating at different levels, more efficient control is anticipated. This is because the regulatory processes occur at different stages: initially at the transcriptional level and subsequently at the translational level [16].

### Phylogenetic enrichment of riboswitches *with tandem adjacent aptamers*

Similar to the tandem riboswitch enrichment analysis, our study examined how organisms from different prokaryotic phyla utilize *riboswitches with adjacent aptamers* to regulate gene expression. Our findings indicate that this enrichment is exclusively observed in *Pseudomonadota* (see Fig. 2, fourth line, and Table S10). This observation aligns with previous reports from our group, which suggest a preference in this phylum for employing translational rather than transcriptional regulatory domains [17]. Specifically, our study found that only 10% of riboswitches in *Pseudomonadota* possess transcription terminators in their regulatory domains, a striking contrast to the nearly 72% observed in *Bacillota* (data not shown). This general tendency for riboswitches in *Pseudomonadota* to lack transcription terminators increases the likelihood of missing regulatory domains after the first riboswitch aptamer. As a result, *tandem adjacent aptamers* emerge as the predominant architecture for contiguous riboswitches in this phylum.

The underlying reason for this low tendency of transcription terminators in the regulatory domains of riboswitches in *Pseudomonadota* remains somewhat unclear, but one plausible explanation may lie in the exceptionally high rate of RNA polymerase synthesis within *Pseudomonadota*. For example, in *Escherichia coli*, RNA polymerase progresses along DNA at speeds of 40 to 80 nt per second, depending on the conditions and specific genes being transcribed [25,26]. This rapid RNA synthesis could limit the time available for riboswitch sensor domains in *Pseudomonadota* to detect their target signals and respond appropriately before RNA polymerase advances too far, rendering the formation of transcriptional terminators ineffective. Consequently, riboswitches with *adjacent aptamers* become the most prevalent type of *tandemly arranged riboswitches* in *Pseudomonadota*.

Unlike *tandemly arranged riboswitches* that sense identical ligands and operate independently, displaying pseudo-cooperative responses, riboswitches with *adjacent aptamers* are proposed to function cooperatively. In these cases, ligand binding to the first aptamer induces a structural change that enhances the affinity of the second aptamer [16].

### Riboswitch families with a significant tendency to be arranged contiguously

**Fig. 3.**
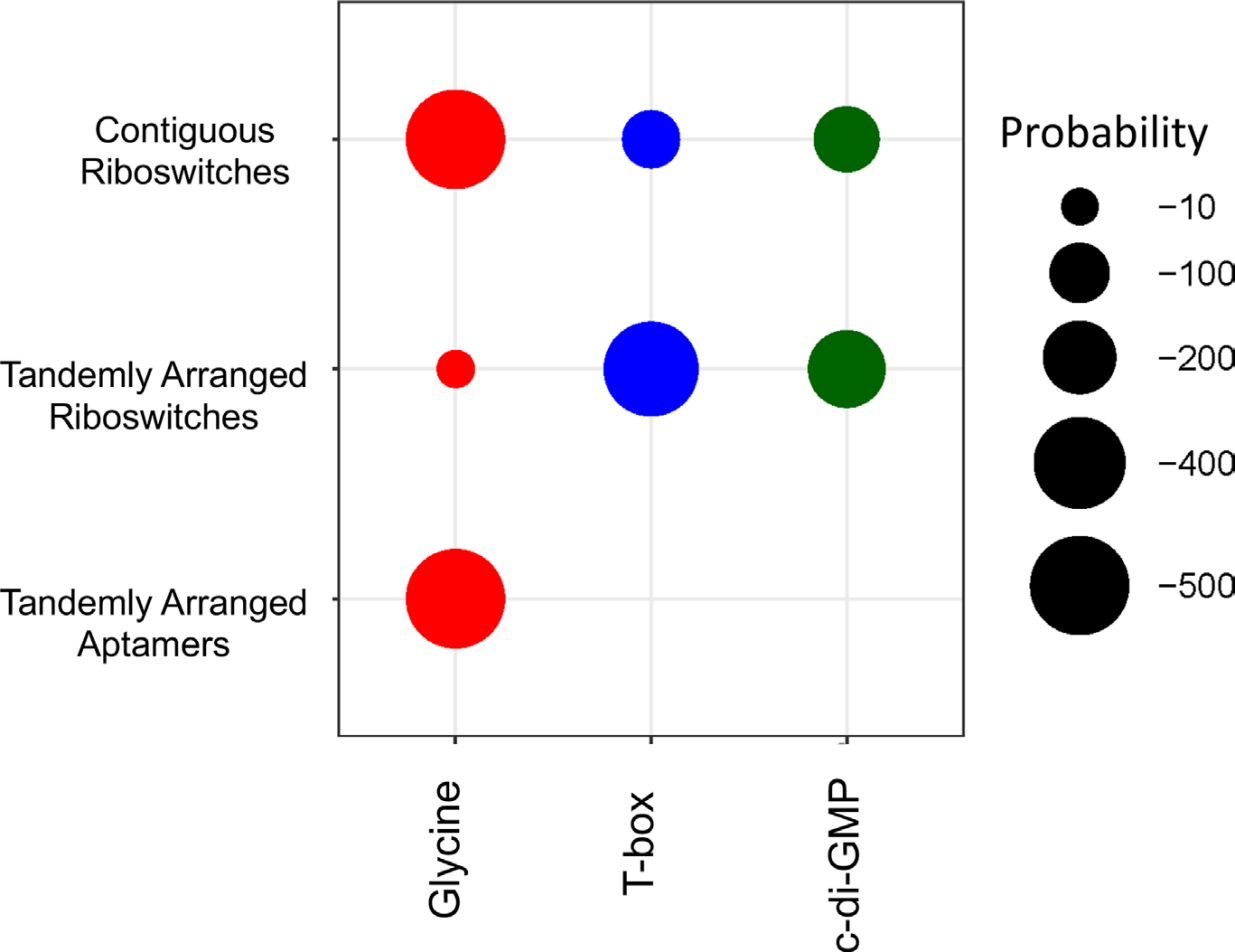
Enrichment of riboswitch families with a significant tendency to be arranged contiguously. The size of the circles represents the *P*-values obtained from our statistical analysis using the hypergeometric distribution (see the ‘Methods’ section). In this context, the *P*-value indicates the probability of observing the same or a greater number of instances of a given riboswitch class in a particular type of arrangement, such as contiguous riboswitches, tandemly arranged riboswitches or those with adjacent aptamers, based on the overall frequency of that arrangement across all riboswitches identified in our study. The input variables used in the hypergeometric test for this analysis are listed in Table 1. Circle colours represent the different riboswitch families that showed statistically significant enrichment.

To ascertain whether all riboswitch families or only specific ones exhibit a tendency to be arranged contiguously, we evaluated their enrichment in the different types of arranged architectures: with *tandemly arranged riboswitches* or *riboswitches with adjacent aptamers*. This evaluation was conducted using hypergeometric distribution analysis (see the ‘Methods’ section). The results of these analyses are summarized in Fig. 3 and Tables S11–S13.

 Of the 27 distinct riboswitch families, only three, glycine, T-box and c-di-GMP, display a pronounced tendency to be *arranged contiguously. Tandem glycine riboswitches* are particularly notable for their enrichment as *adjacent aptamer architectures* (Fig. 3, third line). In contrast, the T-box and c-di-GMP families show significant enrichment in their occurrence as *tandemly arranged riboswitches* (Fig. 3, second line).

 As its name implies, the glycine riboswitch is a regulatory RNA element that controls gene expression in response to the concentration of glycine. Upon binding glycine, the riboswitch undergoes a structural change that can either activate or repress the transcription or translation of associated genes. These genes typically play key roles in glycine metabolism, such as the glycine cleavage system, or in the transport of glycine, enabling the cell to adapt its metabolic processes based on glycine availability. The glycine riboswitches, without any doubt, represent the most extensively studied and characterized family of contiguous riboswitches, with particular emphasis on the chemical, structural and thermodynamic foundations of the cooperativity response mediated by the tertiary interactions between their aptamers, which significantly enhance ligand binding [10,16, 27,30].

Contiguous glycine riboswitches have a significant tendency to be structured as pairs of *tandem adjacent aptamers* followed by a single regulatory platform [10,30]. This could be due to their presence being enriched in *Pseudomonadota*, a phylum where riboswitch expression platforms predominantly favor translational rather than transcriptional regulation. In addition to the cooperative function of their adjacent aptamers, the translational control of most of the contiguous glycine riboswitches tends to be reversible since the three-dimensional structure of their expression platforms might switch depending on the presence of the intracellular glycine concentration [16].

 Conversely, our study revealed that contiguous T-box and c-di-GMP riboswitches exhibit a notable tendency to be organized as *tandemly arranged riboswitches* of two or more contiguous and complete sensor and regulatory domains.

 In the case of the T-box riboswitch, it has been reported to modulate the expression of genes involved in amino acid metabolism, particularly those encoding aminoacyl-tRNA synthetases, amino acid biosynthetic enzymes and amino acid transporters [5,31,33]. It uses uncharged tRNA as a signal molecule. The T-box can fold into two alternative secondary structures: one includes an intrinsic transcription terminator, while the other forms a competing antiterminator structure. When an uncharged tRNA pairs with specific regions of the T-box through codon–anticodon complementarity, it stabilizes the antiterminator structure, preventing the formation of the terminator and promoting full transcriptional readthrough [34]. In contrast, the c-di-GMP riboswitch regulates gene expression in response to intracellular levels of cyclic diguanylate (c-di-GMP), a bacterial second messenger that governs various processes, including motility, virulence and the transition from a free-swimming, motile state to a non-motile, biofilm-associated lifestyle [35,36]. There are two classes of c-di-GMP riboswitches, class I and class II. These classes are not homologous, as they do not share any known sequence or structural similarities.

 Earlier studies have identified genes regulated by *tandemly arranged T-box* [5,37] and c-di-GMP riboswitches [35,36], each composed of one aptamer and one expression platform. As described for this type of *tandem riboswitch arrangement* [16], their regulatory response is expected to function similarly to a digital system with a pseudo-cooperative effect, as the riboswitches act independently yet sense identical ligands. In this configuration, the contiguous T-box and c-di-GMP riboswitches may require both aptamers to bind their respective ligands to promote gene transcription.

### Phylogenetic distribution of riboswitch families with a significant tendency to be arranged in tandem

**Fig. 4.**
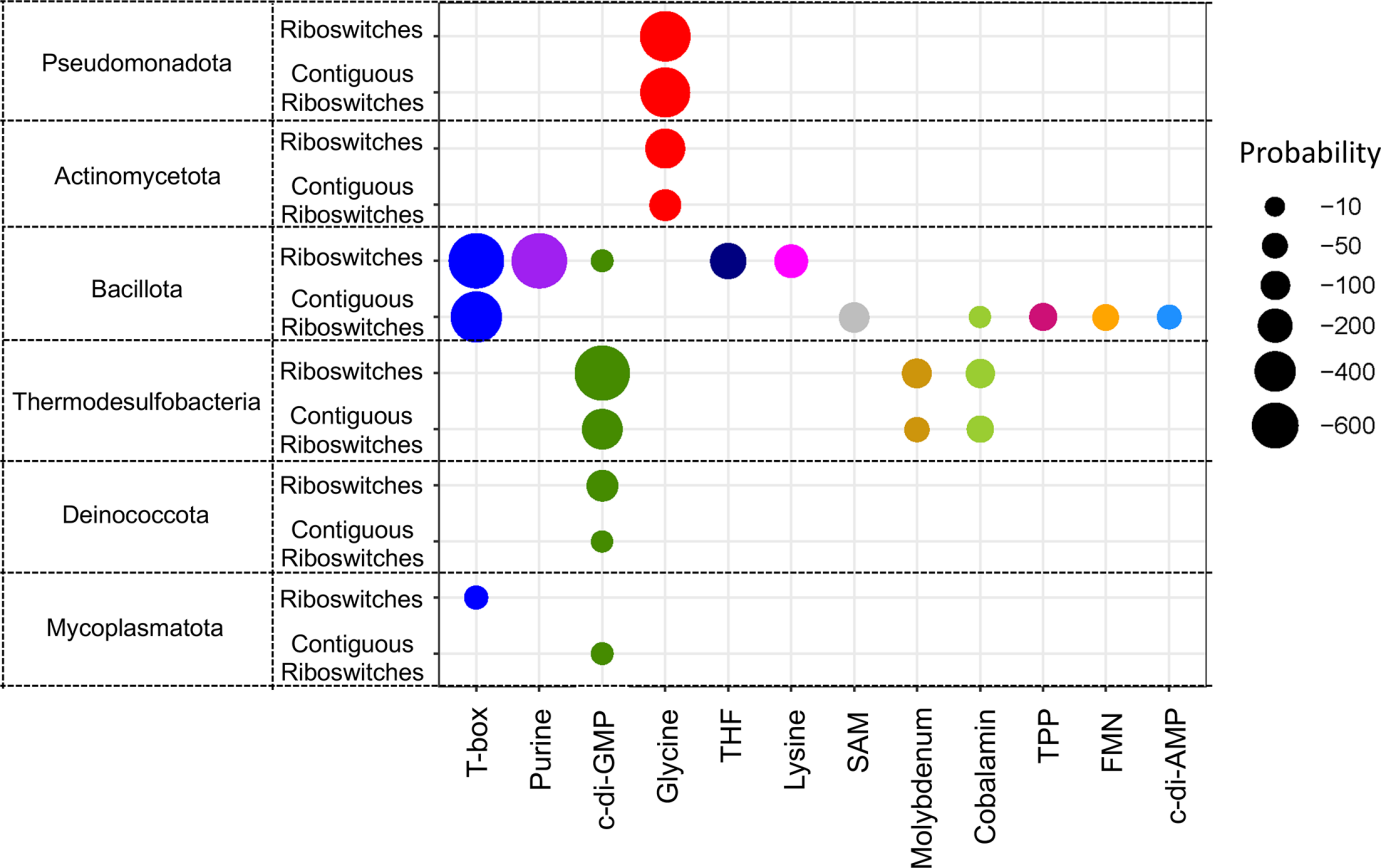
Phylogenetic enrichment patterns for riboswitch families arranged either as single or contiguous units. The size of the circles represents the *P*-values obtained from our statistical analysis using the hypergeometric distribution (see the ‘Methods’ section). In this context, the *P*-value reflects the probability of observing the same or a greater number of instances of a given riboswitch family, arranged either as single or contiguous units, within a specific phylum, based on the overall frequency of that arrangement across all riboswitches identified in that phylum. The variables used in the hypergeometric test are detailed in Table 1. Circle colours correspond to riboswitch families that showed statistically significant enrichment.

 To determine whether the enrichment patterns of glycine, T-box and c-di-GMP riboswitches in contiguous arrangements (Fig. 3 and Table S11) align with broader phylogenetic trends for riboswitches as common regulatory RNA elements (Fig. 2), we applied the hypergeometric test to these riboswitches in their contiguous architecture across different phylogenetic clades. Additionally, we evaluated the phylogenetic enrichment tendencies for these riboswitch families when found in their canonical, single-riboswitch architecture. The results of these analyses are presented in Fig. 4 and Tables S14 and S15.

 The results presented in Fig. 4 indicate that the significant enrichment of *tandemly arranged glycine*, *T-box* and *c-di-GMP riboswitches* across the entire set of prokaryotic organisms is largely driven by their prominent presence in specific phyla. Glycine riboswitches are primarily enriched in *Pseudomonadota* and *Actinomycetota*, T-box riboswitches are predominantly found in *Bacillota*, while c-di-GMP riboswitches are found in *Thermodesulfobacteriota* and *Deinococcota*. This phylum-specific distribution substantially contributes to the overall enrichment patterns observed. Moreover, this enrichment for glycine, T-box and c-di-GMP riboswitches is evident both when these RNA elements regulate in a single-copy manner and when they are arranged contiguously in two or more copies. Conversely, the enrichment of certain riboswitch families in specific phyla, such as THF and lysine riboswitches in *Bacillota*, is predominantly observed when these riboswitches are found as single-copy arrangements, but not when arranged contiguously (Fig. 4 and Tables S14, S15)

### Enrichment of cellular processes regulated by tandemly arranged riboswitches

To identify the key cellular processes regulated by contiguous riboswitches, we analysed the statistical tendencies of orthologous gene groups whose expression is controlled by more than one riboswitch. These gene groups were clustered based on the cellular processes they are involved in. All genes regulated by contiguous riboswitches were annotated according to their orthologous relationships, as outlined in the ‘Methods’ section, and their statistical enrichment was evaluated using the hypergeometric distribution (Table 1). For comparative analysis, we evaluated four distinct cases: (i) enrichment of a COG for regulation by riboswitches (Table S16), (ii) enrichment of a COG within each phylum for regulation by riboswitches (Table S17), (iii) enrichment of a COG for regulation by contiguous riboswitches (Table S18) and (iv) enrichment of a COG within each phylum for regulation by contiguous riboswitches (Table S19).

 The primary cellular processes regulated by contiguous riboswitches are as follows:

*l**-Tryptophan biosynthesis* stands out as the most significantly regulated biochemical pathway by contiguous riboswitches, particularly via *tandemly arranged T-box riboswitches*, which are observed exclusively in *Bacillota*. l-Tryptophan is one of the least abundant amino acids in proteins and the most costly to synthesize in terms of cellular resources and energy [38]. In many bacterial species, the enzymes responsible for tryptophan biosynthesis are co-transcribed within the *trpE-trpG-trpD-trpC-trpF-trpB-trpA* operon, enabling the coordinated expression of these proteins [39].

**Fig. 5.**
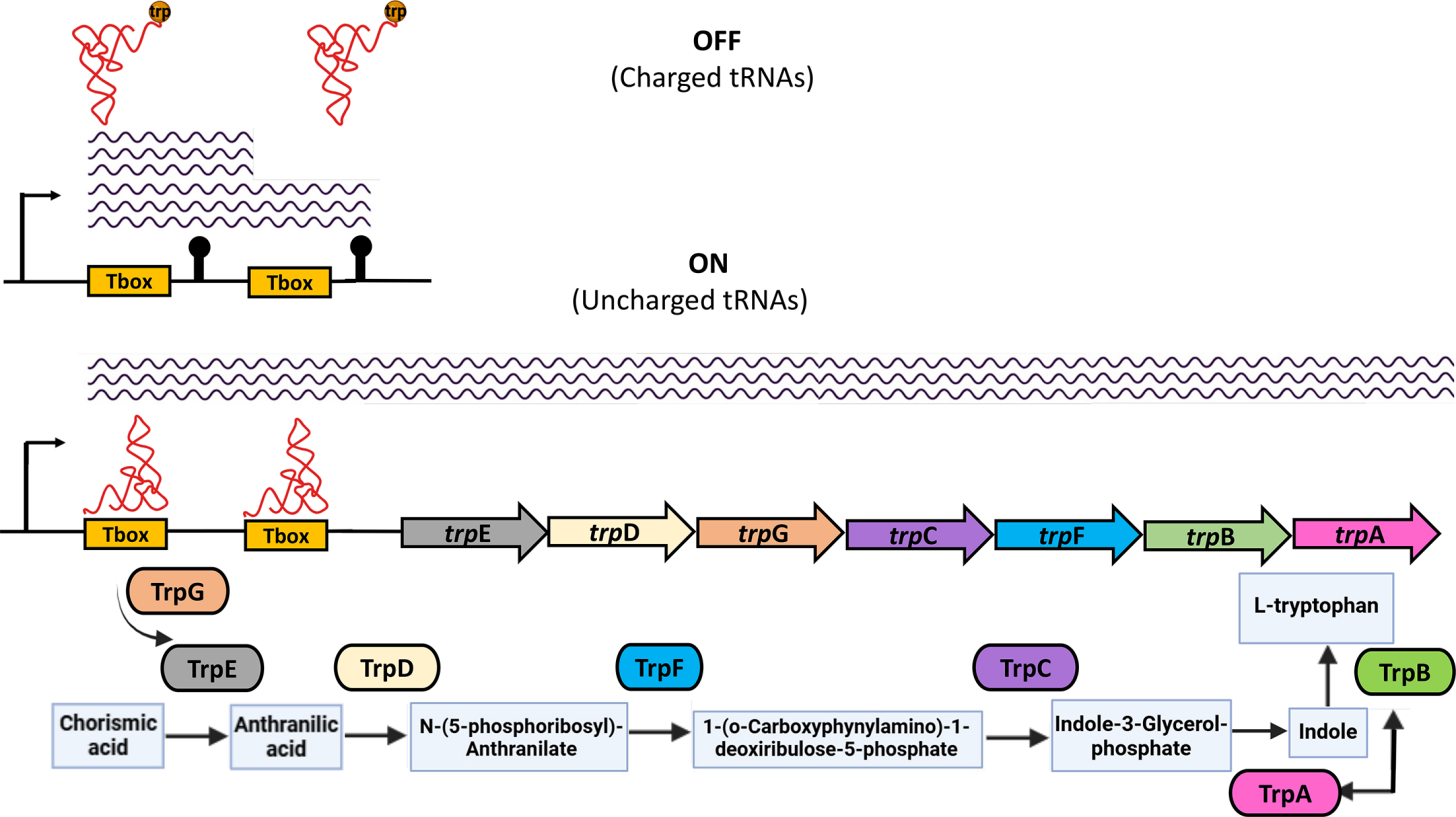
Tandemly arranged T-box riboswitches regulating l-tryptophan biosynthesis. In the figure, operon genes are represented as arrows, and their corresponding enzymes are depicted as colour-coded ovals. Compounds involved as substrates or products in the reactions are shown in blue rectangles. The wavy blue lines represent operon transcripts.

In our study, we found statistically significant enrichments of these COGs regulated by T-box riboswitches, either as single elements (Tables S16, S17) or as contiguous regulatory units (Tables S18, S19), particularly in *Bacillota* (Fig. 5 and Table 2). In a previous study, we documented the evolutionary trends in the operon organization and regulation of genes involved in the tryptophan biosynthetic pathway within this phylum. Some genes are regulated by a single T-box riboswitch, while others are controlled by *tandem copies* of T-box riboswitches [5,37]. In our current study, we identified the exceptional case in *Streptococcus macedonicus*, where the *trp* operon is regulated by three contiguous T-box riboswitches (Table S20).

**Table 2.** Enrichment of COGs regulated by contiguous riboswitches in specific phyla

Phylum	Gene	COG	***P*-value**	Riboswitch	COG description
*Bacillota*	*trpE*	COG0147	1.69E−103	T-box T-box T-box (1%) T-box T-box (99%)	Anthranilate synthase (alpha subunit)
*Bacillota*	*trpG*	COG0512	6.07E−87	T-box T-box T-box (1%) T-box T-box (99%)	Anthranilate synthase (beta subunit)
*Bacillota*	*trpD*	COG0547	6.83E−107	T-box T-box T-box (1%) T-box T-box (99%)	Anthranilate phosphoribosyltransferase
*Bacillota*	*trpC*	COG0134	2.48E−101	T-box T-box T-box (1%) T-box T-box (99%)	Indole-3-glycerol phosphate synthase
*Bacillota*	*trpF*	COG0135	1.36E−87	T-box T-box T-box (1%) T-box T-box (99%)	Phosphoribosylanthranilate isomerase
*Bacillota*	*trpB*	COG0133	1.70E−94	T-box T-box T-box (1%) T-box T-box (99%)	Tryptophan synthase beta chain
*Bacillota*	*trpA*	COG0159	7.13E−99	T-box T-box T-box (1%) T-box T-box (99%)	Tryptophan synthase alpha chain
*Pseudomonadota*	*gcvH*	COG0509	5.62E−43	Glycine Glycine (100%)	Glycine cleavage system H protein (lipoate-binding)
*Pseudomonadota*	*gcvT*	COG0404	9.49E−14	Glycine Glycine (100%)	Glycine cleavage system T protein (aminomethyltransferase)
*Pseudomonadota*	*gcvPA*	COG0403	4.24E−31	Glycine Glycine (100%)	Glycine cleavage system protein P,N-terminal domain
Pseudomonadota	*gcvPB*	COG1003	8.11E−31	Glycine Glycine (100%)	Glycine cleavage system protein P,C-terminal domain
*Actinomycetota*	*glyA*	COG0112	5.22E−48	Glycine Glycine (100%)	Glycine/serine hydroxymethyltransferase
Actinomycetota	*sdhA*	COG1760	6.18E−41	Glycine Glycine (100%)	l-serine dehydratase
*Bacillota*	*leuA*	COG0119	4.64E−54	T-box Guanidine- (24%) T-box T-box (76%)	Isopropylmalate/homocitrate/citramalate synthases
*Bacillota*	*leuB*	COG0473	2.85E−19	T-box T-box (46%) T-box Guanidine- (54%)	Isocitrate/isopropylmalate dehydrogenase
*Bacillota*	*leuC*	COG0065	1.15E−14	T-box Guanidine- (37%) T-box T-box (63%)	3-isopropylmalate dehydratase large subunit
Bacillota	*leuD*	COG0066	6.21E−13	T-box Guanidine- (35%) T-box T-box (65%)	3-isopropylmalate dehydratase small subunit
*Bacillota*	*ilvB*	COG0028	3.56E−50	T-box T-box T-box (2%) T-box Guanidine- (24%) T-box T-box (74%)	Thiamine pyrophosphate-requiring enzymes
*Bacillota*	*ilvN*	COG0440	3.26E−29	T-box T-box T-box (3%) T-box Guanidine- (28%) T-box T-box (68%)	Acetolactate synthase, small (regulatory) subunit
*Bacillota*	*ilvE*	COG0115	6.63E−19	T-box Guanidine- (32%) T-box T-box (68%)	Branched-chain amino acid aminotransferase/4-amino-4-deoxychorismate lyase
*Bacillota*	*ilvC*	COG0059	1.98E−26	T-box Guanidine- (39%) T-box T-box (61%)	Ketol-acid reductoisomerase
*Bacillota*	*ilvD*	COG0129	4.65E−20	T-box Guanidine- (25%) T-box T-box (75%)	Dihydroxyacid dehydratase/phosphogluconate dehydratase
*Bacillota*	*ilvA*	COG1171	2.68E−12	T-box T-box (100%)	Threonine dehydratase
*Bacillota*	*artQ*	COG0765	1.39E−06	T-box T-box (100%)	ABC-type amino acid transport system, permease component
Bacillota	*glnQ*	COG1126	1.15E−059	T-box T-box T-box (6%) T-box T-box (94%)	ABC-type polar amino acid transport system, ATPase component
*Bacillota*	*artP*	COG0834	7.85E−10	T-box T-box T-box (6%) T-box T-box (94%)	ABC-type amino acid transport/signal transduction systems
*Bacillota*	*metQ*	COG1464	1.46E−34	SAM SAM SAM (2%) SAM SAM (42%) T-box T-box (56%)	ABC-type metal ion transport system
*Bacillota*	*metN*	COG1135	5.09E−31	SAM SAM SAM (2%) SAM SAM (41%) T-box T-box (57%)	ABC-type metal ion transport system, ATPase component
*Bacillota*	*metI*	COG2011	1.99E−29	SAM SAM SAM (2%) SAM SAM (42%) T-box T-box (56%)	ABC-type metal ion transport system, permease component
*Bacillota*	*ssuA*	COG0715	1.75E−25	T-box SAM (8%) T-box T-box (12%) SAM SAM (19%) TPP TPP (62%)	Hydroxymethylpyrimidine ABC transporter, substrate-binding component
*Bacillota*	*ssuB*	COG1116	9.41E−24	T-box SAM (8%) T-box T-box (12%) SAM SAM (19%) TPP TPP (62%)	Hydroxymethylpyrimidine ABC transporter, ATPase component
*Bacillota*	*ssuC*	COG0600	5.40E−23	T-box SAM (8%) T-box T-box (12%) SAM SAM (16%) TPP TPP (64%)	Hydroxymethylpyrimidine ABC transporter, transmembrane component
*Bacillota*	*tenA*	COG0819	1.22E−13	Magnesium TPP (4%) TPP TPP (96%)	Thiaminase II
*Bacillota*	*thiE*	COG0352	6.69E−29	TPP TPP TPP (2%) TPP TPP (98%)	Thiamine-phosphate synthase
*Bacillota*	*thiO*	COG0665	1.58E−02	TPP TPP (100%)	Glycine oxidase
*Bacillota*	*thiS*	COG2104	2.23E−14	TPP TPP (100%)	Sulphur carrier protein thiamine biosynthesis
*Bacillota*	*thiG*	COG2022	1.34E−13	TPP TPP (100%)	Thiazole synthase
*Bacillota*	*thiF*	COG0476	6.43e−13	TPP TPP (100%)	Thiazole biosynthesis adenylyltransferase
*Bacillota*	*thiD*	COG0351	2.03e−22	TPP TPP TPP (3%) TPP TPP (97%)	Hydroxymethylpyrimidine kinase/phosphomethylpyrimidine kinase
*Bacillota*	*thiM*	COG2145	8.40e−13	TPP TPP TPP (4%) TPP TPP (96%)	Hydroxyethylthiazole kinase
*Bacillota*	*thiC*	COG0422	6.99E−12	TPP TPP TPP (8%) TPP TPP (92%)	Phosphomethylpyrimidine synthase
*Bacillota*	*ribE*	COG0307	4.75e−30	FMN FMN FMN (13%) FMN FMN (87%)	Riboflavin synthase alpha chain
*Bacillota*	*ribB*	COG0108	1.39e−27	FMN FMN FMN (13%) FMN FMN (87%)	3,4-Dihydroxy-2-butanone4-phosphate synthase
*Bacillota*	*ribH*	COG0054	1.18e−29	FMN FMN FMN (13%) FMN FMN (87%)	Riboflavin synthase beta-chain
*Bacillota*	*hom1*	COG0460	1.866E−16	Cobalamin SAM SAM (9%) SAM SAM (30%) T-box T-box (61%)	Homoserine dehydrogenase
*Bacillota*	*thrC*	COG0498	1.18E−10	T-box T-box (100%)	Threonine synthase
*Bacillota*	*thrB*	COG0083	3.00E−08	T-box T-box (100%)	Homoserine kinase
*Bacillota*	*thrS*	COG0441	4.92E−51	T-box T-box T-box (13%) T-box T-box (87%)	Threonyl-tRNA synthetase
*Bacillota*	*yebA*	COG1305	1.08E−34	c-di-GMP-I c-di-GMP-I c-di-GMP-I (6%) c-di-GMP-I c-di-GMP-I (94%)	Transglutaminase-like enzymes, putative cysteine proteases
*Bacillota*	*amhX*	COG1473	1.63E−20	SAM SAM (40%) T-box T-box (60%)	Metal-dependent amidase/aminoacylase/carboxypeptidase

**Fig. 6.**
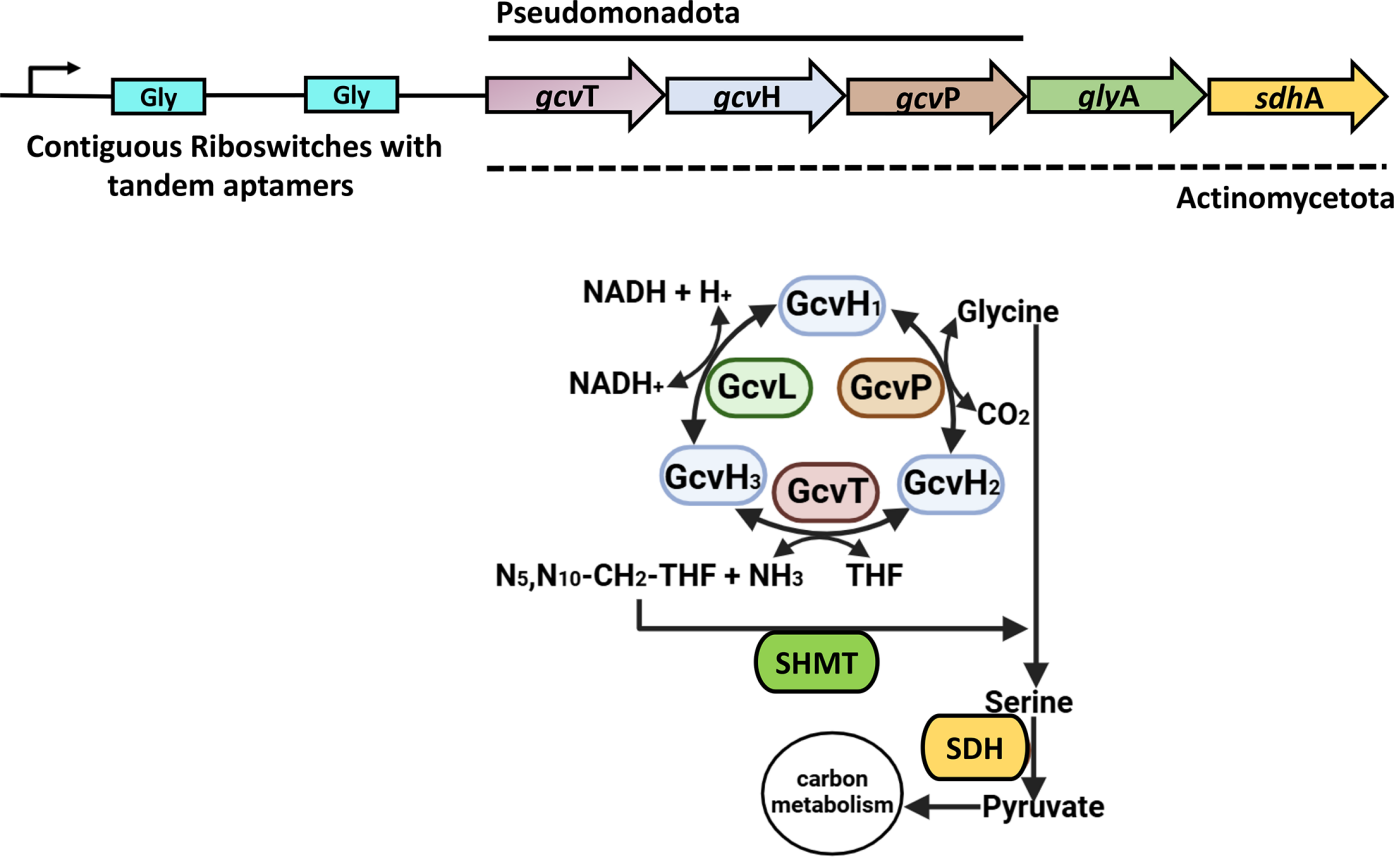
Tandemly arranged glycine riboswitches regulating the glycine cleavage system. In the figure, operon genes are depicted as arrows, and their corresponding enzymes are shown as ovals, with matching colours illustrating the relationship between each gene and its encoded protein. The solid line above the operon delimits the glycine cleavage genes transcribed as a polycistronic unit in *Pseudomonadota*, while the dashed line below delimits a larger operon commonly found in *Actinomycetota*.

 The *glycine cleavage system* is the second most significant biochemical process regulated by contiguous riboswitches identified in our analysis. This process is mediated by enzymes encoded by the *gcvTHP* operon, which is regulated by adjacent glycine riboswitches, particularly in *Pseudomonadota*, allowing adaptation to excess glycine (Fig. 6, Tables 2 and S16 to S19) [24,40]. GcvP decarboxylates glycine, generating a methylamine group that GcvH transfers to GcvT, forming 5,10-methylene-THF, a critical intermediate in one-carbon metabolism. This pathway supports energy production and mitigates glycine toxicity [41,43].

 In *Pseudomonadota*, we found significant enrichments of *gcvH*, *gcvT* and the N-terminal (*gcvPA*) and C-terminal (*gcvPB*) domains of *gcvP*. Operon analysis revealed that 98% of glycine cleavage systems regulated by *contiguous glycine riboswitches* include *gcvT* and *gcvH*, while only 48% include the entire *gcvTHP* cluster (Table S20). In some organisms, *gcvPA* and *gcvPB* are encoded separately but typically transcribed as part of the *gcvT-gcvH-gcvPA-gcvPB* operon (Table S20), similarly regulated by tandem glycine riboswitches [44].

In *Actinomycetota*, we found that the *gcvT-gcvH-glyA-sdhA* operon is regulated by *contiguous glycine riboswitches* in various *Streptomyces* species, including *Streptomyces huasconensis*, *Streptomyces alboniger*, *Streptomyces atratuspratensis*, *Streptomyces formicae*, *Streptomyces griseorubiginosus*, *Streptomyces huasconensis* and *Streptomyces koyangensis*, among others (Table S20). This operon encodes proteins for glycine degradation (GcvT and GcvH), serine-glycine interconversion (GlyA) and energy production (SdhA) [45,46]. Tandem riboswitches regulate serine conversion to pyruvate when glycine is scarce, supporting energy production and ensuring balanced amino acid levels within the cell.

*The biosynthesis of branched-chain amino acids (BCAAs)*, leucine, isoleucine and valine, is another cellular process identified in our study as being enriched in the regulation by *tandemly arranged riboswitches*, exclusively in *Bacillota* (Tables 2 and S19). This pathway begins with shared initial steps using pyruvate as a common precursor before diverging into distinct branches specific to each amino acid. The genes involved in BCAA biosynthesis are typically organized into two operons, *leuA-leuB-leuC-leuD* and *ilvB-ilvN-ilvE-ilvC-ilvD-ilvA*. However, frequent variations from this canonical operon organization are observed across different organisms, and in many cases, *ilvD* and *ilvE* are monocistronic (Table S20) [5,47, 48].

The *leu* operon encodes enzymes for leucine synthesis, including isopropylmalate synthase (LeuA), dehydrogenase (LeuB) and isomerase subunits (LeuC and LeuD). On the other hand, the *ilv* operon encodes enzymes for all BCAAs, such as acetohydroxyacid synthase (IlvB and IlvN), transaminase (IlvE), reductoisomerase (IlvC), dehydratase (IlvD) and threonine deaminase (IlvA), linking threonine metabolism to the biosynthesis of branched-chain amino acids that are important for protein synthesis, energy production and various metabolic processes [5,47, 48].

**Fig. 7.**
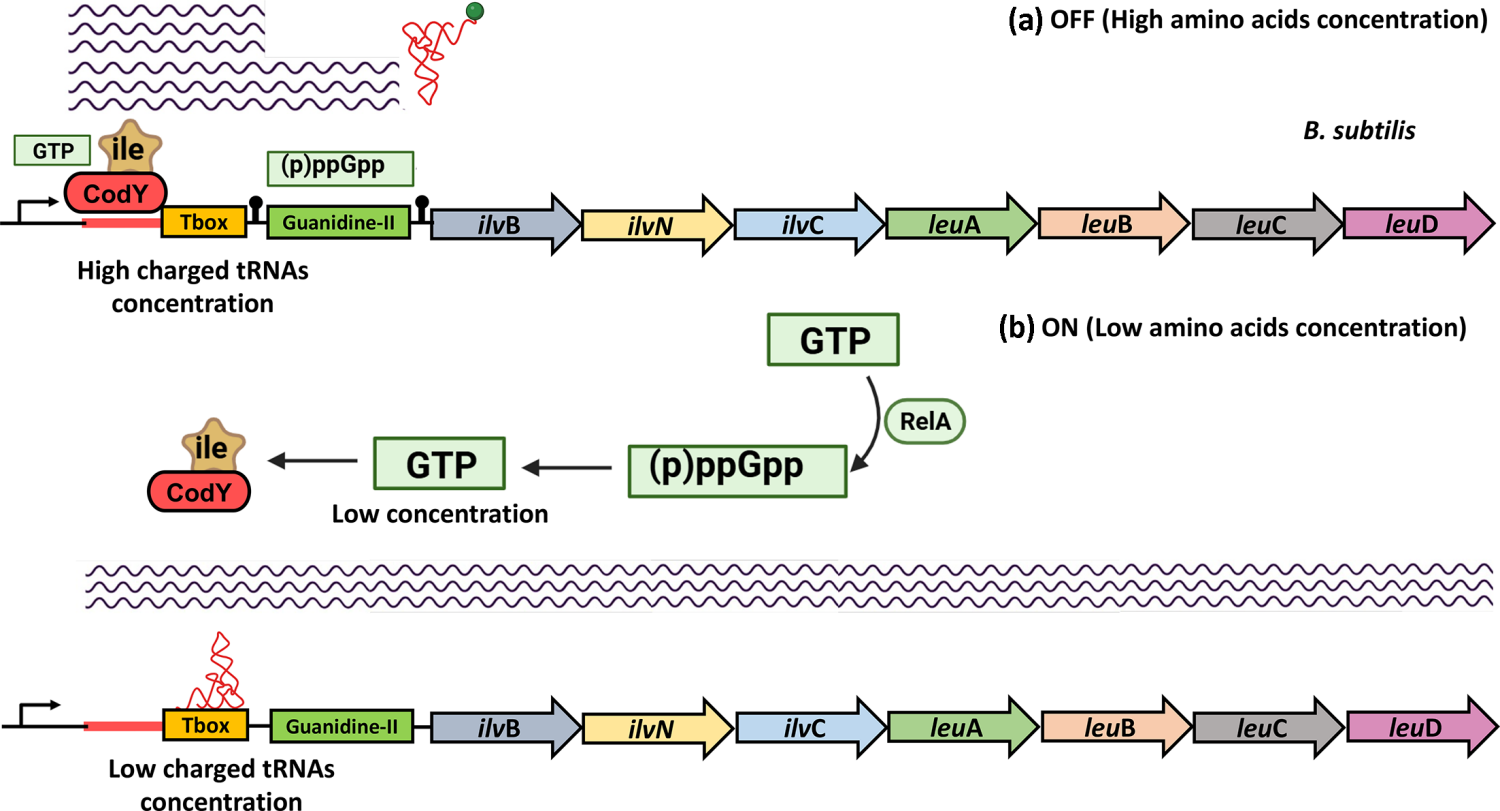
Coordinated regulation by the transcription factor CodY and tandemly arranged T-box and guanidine riboswitches in the biosynthesis of the branched-chain amino acids. In the figure, operon genes are depicted as arrows, and their encoded enzymes are illustrated as ovals, with matching colours used to emphasize the correspondence between each gene and its protein product. (**a**) When the T-box and guanidine riboswitches adopt regulatory conformations that include Rho-independent transcriptional terminators, transcription is prematurely halted, resulting in short transcripts. (**b**) In contrast, when these riboswitches fold into antiterminator structures, they permit full-length transcription of the operon.

The regulation of the BCAA biosynthetic pathway by *tandemly arranged riboswitches* is primarily mediated by contiguous T-box riboswitches (Tables 2, S18 and S19). In this case, the expression of BCAA biosynthetic operons requires a substantial decrease in the levels of charged tRNA, as two uncharged tRNA molecules are needed to prevent premature transcription termination at either of the T-box riboswitch regulatory platforms. To a lesser extent, we also identified instances where the BCAA biosynthetic operon is preceded by tandemly arranged T-box and guanidine riboswitches (Fig. 7). It has been reported that the guanidine riboswitch responds to the presence of guanidine, a toxic byproduct that can accumulate under certain conditions. This riboswitch typically regulates genes involved in guanidine detoxification and export [49]. We speculate that the dual regulation of BCAA biosynthesis operons by both the T-box and guanidine riboswitches might ensure operon expression when amino BCAAs are scarce, promoting their synthesis. Additionally, the guanidine riboswitch may provide an extra layer of control to fine-tune this process under stress conditions, such as when guanidine is present.

Interestingly, while in some organisms the genes involved in the biosynthesis of BCAAs are regulated by contiguous T-box riboswitches, in some others, this regulation is performed by the additive interplay of the T-box riboswitch and different transcription factors (Table S20). For instance, in the model organism *Bacillus subtilis*, the *ilv* and *leu* genes are organized into a single operon, *ilvBNC-leuABCD*, which is regulated by a single T-box riboswitch that senses uncharged leucine tRNA [5] and the regulatory activity of the CodY DNA-binding protein, which represses the *ilvB* promoter in response to the availability of isoleucine and valine, in such a way that the expression of the *ilvBNC-leuABCD* operon responds in accordance with the intracellular levels of all three branched-chain amino acids (Fig. 7) [5]. Additionally, this operon is also regulated by TnrA, which under conditions of nitrogen scarcity is activated and binds to specific DNA sequences, promoting the expression of genes involved in nitrogen assimilation and metabolism [50,51] and by CcpA, which is activated in the presence of glucose or other preferred carbon sources and overrides repression by CodY, a regulator that responds to nutrient availability [52,53].

*The biosynthesis of ABC-type transport systems* was found to be statistically enriched for regulation by *tandemly arranged riboswitches*, observed exclusively in *Bacillota* (Tables 2 and S19). Based on the classification of their corresponding COGs, we identified three primary groups of transport systems (Table S20).

The first group of ABC-type transport systems encompasses amino acid transport and signal transduction systems. Key representatives of this group are typically co-transcribed within the *artQ-glnQ-artP* operon (Table S20).

ArtQ and ArtP function together as part of a protein complex that regulates amino acid transport. ArtQ acts as a sensor kinase, detecting the presence of arginine and other amino acids, and modulates ArtP’s activity to adjust transport levels accordingly. ArtP, a response regulator, interacts with ArtQ to control the expression of genes involved in amino acid transport and metabolism [54]. In contrast, GlnQ serves as a sensor kinase in nitrogen metabolism, sensing the availability of nitrogen sources and adjusting the activity of its associated response regulator to manage the expression of genes involved in nitrogen assimilation.

In most cases where the genes encoding these proteins are regulated by contiguous riboswitches, these riboswitches consist of a pair of similar T-box elements. Notably, we identified exceptional instances where up to three contiguous T-box riboswitches regulate the *artQ-glnQ-artP* operon (Table S20).

The second group of ABC-type transport systems is associated with metal ion uptake, efflux and intracellular distribution, ensuring that cells maintain the appropriate levels of essential metal ions. Metal ions such as iron, zinc, copper and manganese play crucial roles as cofactors in enzymatic reactions, are vital components of electron transport and contribute to the structural stability of proteins and nucleic acids [55]. These transport systems are typically encoded within the *metN-metI-metQ* operon [56]. In nearly one-third of the cases, this operon also includes the *amhX* gene, which encodes a metal-dependent enzyme with amidase, aminoacylase or carboxypeptidase activities that require a metal ion as a cofactor (Table S20). This dependency may explain the inclusion of the *amhX* gene in operons that code for ABC-type metal ion transporters. The genes in this group are primarily regulated by either a pair of tandem T-box or SAM riboswitches. In exceptional cases, we identified up to three copies of SAM riboswitches arranged in tandem (Tables 2 and S20).

The SAM riboswitch primarily regulates genes involved in the biosynthesis and transport of methionine, cysteine, SAM and related compounds. Elevated intracellular levels of SAM can indicate a high capacity for methylation, prompting the cell to ensure that adequate metal ions are available to support the activity of SAM-dependent enzymes. When SAM concentrations rise, the SAM riboswitch can modulate the expression of ABC-type transporters, facilitating the uptake of essential metal ions necessary for sustaining methylation and other SAM-related processes.

In the context of ABC-type metal ion transport systems regulated by tandem T-box riboswitches, it is crucial to recognize that some enzymes involved in amino acid biosynthesis require metal ions as cofactors. For instance, aminoacyl-tRNA synthetases, responsible for charging tRNAs with amino acids, often depend on metal ions for their catalytic function [57,58]. A deficiency in these metal ions could indirectly hinder amino acid metabolism by impairing enzyme activity. In this scenario, the T-box riboswitch might regulate genes responsible for metal ion transport to ensure that sufficient metal ions are available for amino acid biosynthesis. For example, when uncharged tRNAs accumulate, indicative of a shortage of specific amino acids, the T-box riboswitch could upregulate metal ion transport, ensuring that metal-dependent enzymes can operate effectively.

The third group of ABC transport systems that demonstrates statistically significant enrichment for regulation by contiguous riboswitches is comprised of hydroxymethylpyrimidine (HMP) ABC transporters. Thiamine pyrophosphate, the active form of vitamin B1, serves as an essential coenzyme synthesized in bacteria by coupling two critical components: HMP and hydroxyethylthiazole (HET). These components contribute to the formation of the pyrimidine and thiazole rings, which are subsequently combined to produce thiamine. HMP ABC transporters facilitate the uptake of hydroxymethylpyrimidine into the cell [59]. These transport systems consist of a multi-component protein complex, including SsuA (substrate-binding protein), which exhibits a high affinity for hydroxymethylpyrimidine and presents it to the transport system; SsuC, which forms the channel for substrate transport across the cell membrane; and CmpD, which hydrolyses ATP to provide the necessary energy for the transport process. Typically, the genes encoding these proteins are co-transcribed within an operon associated with thiamine biosynthesis: *tenA-cmpD-ssuC-ssuA-thiE-thiO-thiS-thiG-thiF-thiD* (Table S20).

*Thiamin biosynthesis* is another biological process that is significantly regulated by contiguous riboswitches (Tables 2, S18 and S19). The pathway begins with ThiC, which catalyses the synthesis of 4-amino-5-hydroxymethyl-2-methylpyrimidine phosphate (HMP-P) from 5-aminoimidazole ribonucleotide for the pyrimidine moiety. ThiG uses sulphur carried by ThiS to produce 4-methyl-5-(2-hydroxyethyl)thiazole phosphate (HET-P) for the thiazole moiety. ThiD converts 4-amino-5-hydroxymethyl-2-methylpyrimidine (HMP) into HMP-P and then into HMP-pyrophosphate (HMP-PP). ThiE combines HMP-PP and HET-P to form thiamine monophosphate, later phosphorylated into active TPP. ThiM phosphorylates 4-methyl-5-(2-hydroxyethyl)thiazole (HET) to HET-P. In some bacteria, ThiO generates glyoxylate for thiazole formation, and TenA breaks down thiamine or its derivatives for reuse in the pathway [60].

As previously mentioned, the regulation of the *tenA-cmpD-ssuC-ssuA-thiE-thiO-thiS-thiG-thiF-thiD* operon, which governs the biosynthesis and transport of thiamine, is characterized by its complexity, primarily due to control by *contiguous riboswitches* (Tables 2, S18 and S19). Initially, this operon is regulated by tandem TPP riboswitches. Additionally, regulation is influenced by tandem SAM riboswitches and tandem T-box riboswitches, along with, to a lesser extent, the combination of T-box and SAM riboswitches (Tables 2 and S20).

The TPP riboswitch is a regulatory RNA element that detects TPP, the active form of vitamin B1, and modulates the expression of genes involved in thiamine biosynthesis and transport. When TPP levels are adequate, this riboswitch represses its target genes, conserving cellular resources by preventing unnecessary thiamine production or uptake. Our observations of contiguous TPP riboswitches regulating the operon involved in thiamine synthesis and transport support this well-established regulatory mechanism [13].

In another instance, we discovered that this operon is regulated in *Bacillota* by *tandemly arranged SAM riboswitches*, which sense S-adenosylmethionine (Tables 2 and S19). SAM is a vital methyl donor involved in numerous biochemical processes, including the methylation of nucleic acids, proteins and lipids. Thiamine, in its active form (TPP), acts as a cofactor in several key metabolic reactions, such as those in the citric acid cycle and the pentose phosphate pathway. The biosynthesis of both SAM and TPP is energetically demanding and tightly regulated to maintain cellular homeostasis. We hypothesize that elevated SAM levels may indicate a metabolically favourable state, thereby diminishing the necessity for expression of the *tenA-cmpD-ssuC-ssuA-thiE-thiO-thiS-thiG-thiF-thiD* operon and further TPP production.

Additionally, we found that the operon responsible for thiamine biosynthesis could be regulated by tandem T-box riboswitches. One plausible explanation for this regulation is that when amino acid levels are low, the cell must enhance biosynthetic activities to restore equilibrium. Given that thiamine is a crucial cofactor in various metabolic pathways, including those involved in amino acid processing, the cell needs to maintain adequate thiamine levels, even amid amino acid scarcity. Consequently, T-box regulation ensures that when intracellular amino acid concentrations decrease, transcription of the *tenA-cmpD-ssuC-ssuA-thiE-thiO-thiS-thiG-thiF-thiD* operon is activated, facilitating thiamine production or uptake from the environment to sustain critical metabolic functions. Moreover, we identified a statistically significant enrichment of COGs encoded in the *tenA-cmpD-ssuC-ssuA-thiE-thiO-thiS-thiG-thiF-thiD* operon that are also regulated by *tandemly arranged riboswitches* (Tables 2 and S20). This further underscores the regulatory complexity surrounding thiamine biosynthesis in *Bacillota*.

Our study also identified *riboflavin biosynthesis* as a metabolic process statistically enriched for regulation by contiguous riboswitches, specifically through the presence of double or triple FMN riboswitches arranged in tandem (Tables 2, S17 and S18). This riboswitch binds FMN to control gene expression in riboflavin (vitamin B2) biosynthesis and uptake [24].

In our study, we identified a statistically significant enrichment of proteins involved in riboflavin biosynthesis (Tables 2, S16 and S19). These proteins are typically encoded within the conserved *ribE-ribB-ribH* operon. RibB catalyses the conversion of ribulose-5-phosphate into 3,4-dihydroxy-2-butanone-4-phosphate, RibH produces 6,7-dimethyl-8-ribityllumazine and RibE synthesizes riboflavin. Riboflavin serves as a precursor for the essential cofactors flavin adenine dinucleotide and FMN, which are crucial for biological redox reactions [61,62].

**Fig. 8.**
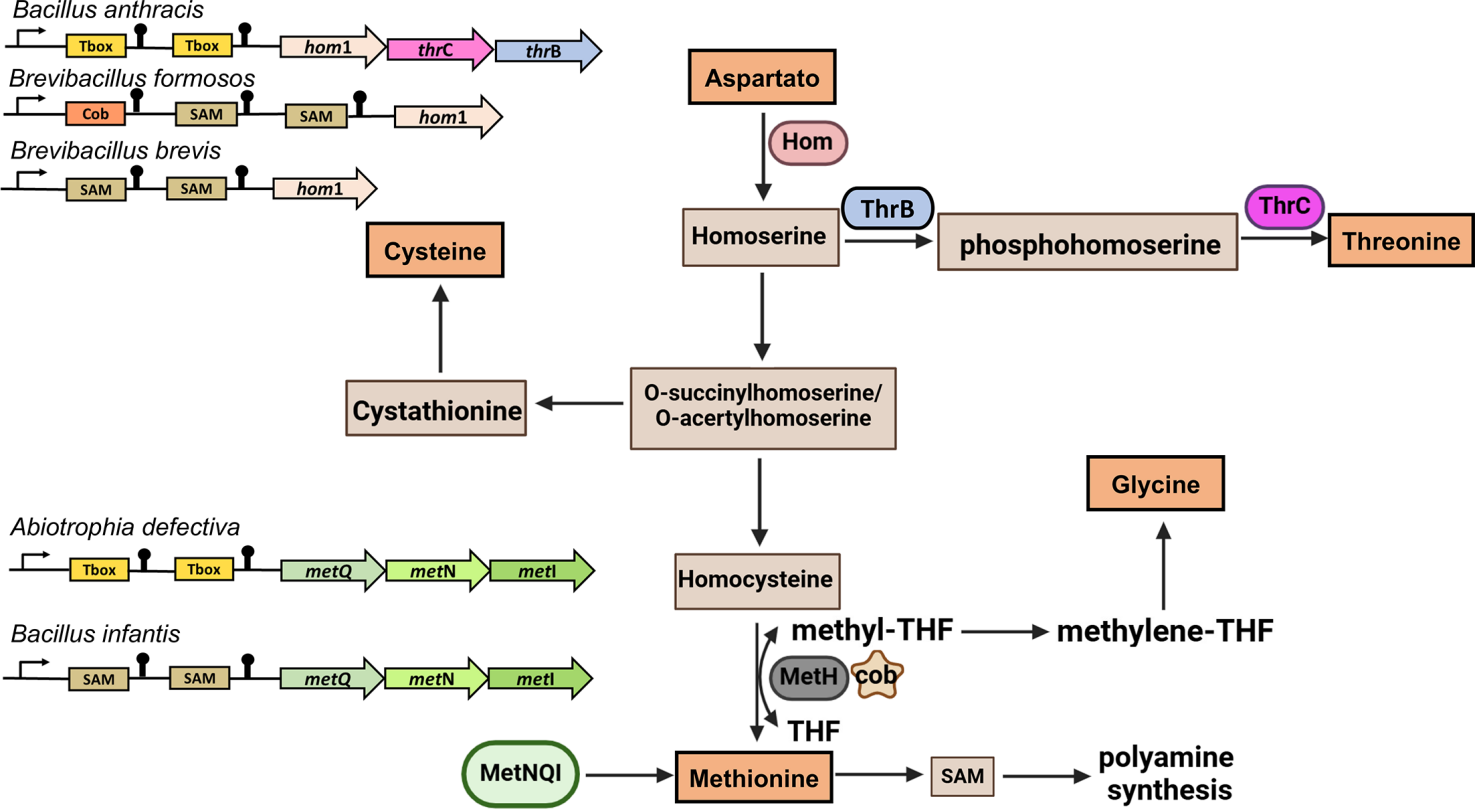
Tandemly arranged T-box, SAM and cobalamin riboswitches regulating the biosynthesis of the aspartate family of amino acids. In the figure, genes within the operon are depicted as directional arrows, while the enzymes they encode are represented by ovals distinguished by colour.

*The biosynthesis of the aspartate family of amino acids*, including diaminopimelate, lysine, methionine and threonine, was identified in our study as statistically significantly regulated by contiguous riboswitches (Fig. 8). These amino acids derive their carbon atoms from l-aspartate, making them part of a complex metabolic network with shared intermediates that branch into distinct biosynthetic pathways. This interconnected process efficiently generates various essential biomolecules [63]. In *Bacillota*, we identified significant enrichment of the conserved *hom1-thrC-thrB* operon regulated by tandem riboswitches (Fig. 8, Tables 2, S18 and S19). Notably, this type of regulation was also observed when the *hom1* gene is transcribed as a monocistronic unit.

Hom1 (homoserine dehydrogenase) is essential in the biosynthesis of threonine, methionine and isoleucine, catalysing the reduction of aspartate semialdehyde to homoserine [44,64]. ThrB (homoserine kinase) phosphorylates homoserine to form O-phosphohomoserine, a key intermediate for threonine and methionine. ThrC (threonine synthase) converts O-phosphohomoserine into threonine. Our study identified complex regulation of the *hom1* gene, mediated by various contiguous riboswitch arrangements, including a pair of contiguous T-box riboswitches, a pair of contiguous SAM riboswitches and a cobalamin riboswitch followed by a pair of contiguous SAM riboswitches (Fig. 8, Tables 2 and S20). These diverse regulatory mechanisms highlight the intricate control of *hom1* expression through different combinations of contiguous riboswitches.

In the first instance, the regulation of *tandemly arranged T-box riboswitches* could ensure that the *hom1* monocistronic unit or the *hom1-thrC-thrB* operon would be produced when the intracellular concentrations of amino acids like threonine and methionine are low. The regulation of the *hom1* monocistronic unit by *tandemly arranged SAM riboswitches* could be explained considering that SAM is a key metabolite in the methionine cycle. Since homoserine dehydrogenase is involved in the biosynthesis of homoserine, a precursor to methionine, its regulation can directly influence methionine levels. Additionally, the presence of a cobalamin riboswitch preceding a pair of contiguous SAM riboswitches reflects the crucial role of cobalamin as a cofactor for methionine synthase, which converts homocysteine to methionine (Fig. 8). As homoserine dehydrogenase contributes to homoserine production, its activity directly impacts methionine synthesis. Consequently, fluctuations in cobalamin levels, which signal methionine requirements, can modulate the regulation of homoserine dehydrogenase. This integration of cobalamin and SAM riboswitches exemplifies how the cell manages methionine biosynthesis in response to metabolic demands.

**Fig. 9.**
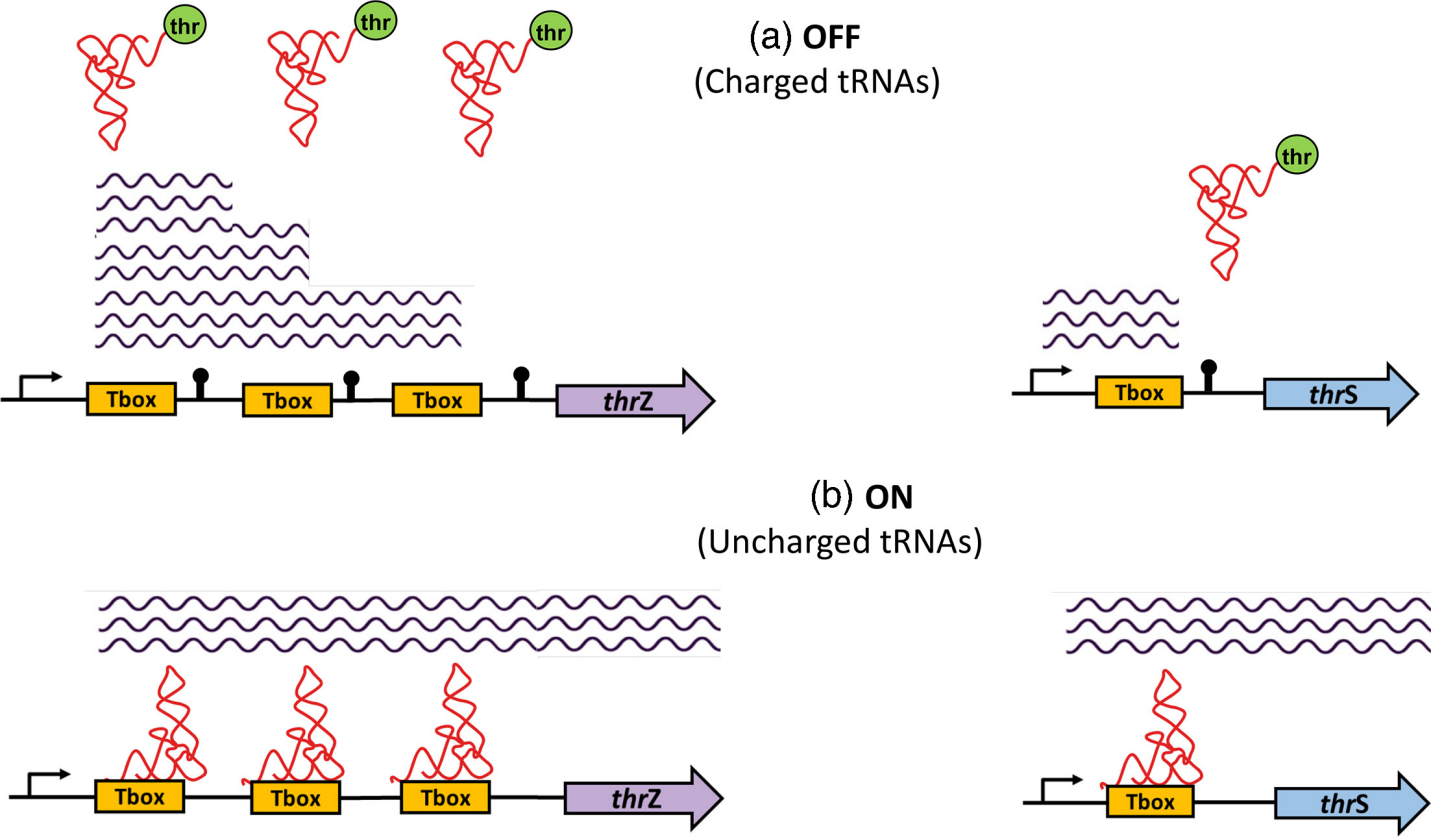
Regulation of aminoacyl-tRNA synthetase genes by T-box riboswitches in *Bacillota*. In the figure, operon genes are represented as arrows. (**a**) In *Bacillota*, threonyl-tRNA synthetase genes are frequently regulated by two or even three tandemly arranged T-box riboswitches. (**b**) In contrast, all other aminoacyl-tRNA synthetase genes in *Bacillota* are typically regulated by a single T-box riboswitch.

*Aminoacylation of threonine by the threonyl-tRNA synthetase* is another process statistically significantly regulated in *Bacillota* by *tandemly arranged* T-box riboswitches (Fig. 9, Tables 2, S18 and S19). These enzymes catalyse the attachment of amino acids to their corresponding tRNA molecules, a key step in protein synthesis. This process ensures accurate codon–anticodon pairing during translation. The T-box riboswitch system exemplifies one of the earliest known mechanisms of riboswitch-mediated gene expression regulation [31,33].

Our study identified that among all aminoacyl-tRNA synthetases (aaRS) responsible for charging the 20 standard amino acids, only threonyl-tRNA synthetase (ThrS), which charges threonine to its cognate tRNA, is regulated by tandemly arranged T-box riboswitches. In contrast, other aaRS enzymes are primarily regulated by single riboswitch copies (Tables S16 and S17). Notably, in 87% of cases, ThrS regulation involves a pair of T-boxes, while 13% involve three T-box riboswitches, as observed in species like *Priestia filamentosa*, *Metabacillus dongyingensis*, *Paenibacillus lutimineralis* and *Bacillus mycoides* (Table S20).

Previous analyses from our group have uncovered multiple instances of T-box regulation across nearly all aaRS enzymes responsible for charging the 20 standard amino acids. In most cases, the genes encoding aaRS are transcribed as monocistronic units [5]. In this study, we identify two novel types of polycistronic operon organization that include aaRS genes regulated by tandem T-box riboswitches (Tables 2 and S20). The first operon, *thrS-infC-rpmI-rplT*, encodes threonyl-tRNA synthetase, translation initiation factor 3, ribosomal proteins L35 and L20 and rRNA methylases. This operon organization is conserved across multiple *Clostridium* species, including *Clostridium carboxidivorans*, *Clostridium drakei*, *Clostridium fermenticellae* and *Clostridium scatologenes*. The second operon includes genes encoding threonyl-tRNA synthetase, an ArsR family transcriptional regulator and a major facilitator superfamily (MFS) transporter. This regulatory arrangement has been identified in *Bacillus* species such as *Bacillus* sp. BH072, *Bacillus* sp. SDLI1 and *Bacillus siamensis*. While the role of threonyl-tRNA synthetase in aminoacylation is well-established, the functional significance of the other operon components, particularly the ArsR transcriptional regulator and MFS transporter, remains uncertain. The lack of characterization of the regulatory targets of the ArsR family complicates efforts to propose a clear hypothesis regarding the biological role of these operons, suggesting avenues for future investigation into their regulatory and functional implications.

## Conclusions

While the presence of contiguously positioned riboswitches has been documented for specific families and their molecular mechanisms characterized and compared to logic gates, their distribution and relative prevalence across various phylogenetic groups have largely remained unexplored. In this study, we conducted statistical analysis primarily focusing on previous reports examining the transcription rates of RNA polymerase in different phylogenetic clades. Among other relevant insights, our study makes two significant contributions. First, we demonstrate that tandemly arranged riboswitches, which use Rho-independent transcriptional terminators as expression platforms for the first riboswitch, are more prevalent in *Bacillota*. This prevalence is likely due to the slower transcription rate in *Bacillota* compared to other phylogenetic groups. In contrast, we found that *Pseudomonadota*, with faster transcription rates, tend to have an enrichment of tandem sensor domains, particularly those associated with glycine detection. Second, our comprehensive statistical analysis of all known riboswitches in a set of complete and non-redundant genome sequences offers a clear overview of the key cellular processes that require tight regulation by these RNA regulatory elements when arranged contiguously.

## Supplementary material

10.1099/mgen.0.001496 Uncited Supplementary Material 1.

10.1099/mgen.0.001496 Uncited Supplementary Material 2.

## Abbreviations

aaRS: aminoacyl-tRNA synthetases
BCAAs: branched-chain amino acids
c-di-GMP: cyclic dimeric guanosine monophosphate
COG: Cluster of Orthologous Group
FMN: flavin mononucleotide
GA: gathering
Gly: glycine
HET: hydroxyethylthiazole
HET-P: 4-methyl-5-(2-hydroxyethyl)thiazole phosphate
HMP: hydroxymethylpyrimidine
HMP-P: 4-amino-5-hydroxymethyl-2-methylpyrimidine phosphate
HMP-PP: HMP-pyrophosphate
KEGG: Kyoto Encyclopedia of Genes and Genomes
MFS: major facilitator superfamily
SAM: *S*-adenosyl methionine
ThrS: threonyl-tRNA synthetase
TPP: thiamine pyrophosphate
