# Supplementary figures and images for: Variation in phylogenetic tendencies of contiguous riboswitches
Source: Microb Genom. 2025 Sep 17;11(9):001496. doi: 10.1099/mgen.0.001496 (PMC12452182; doi:10.1099/mgen.0.001496)

Supplementary Figure S1

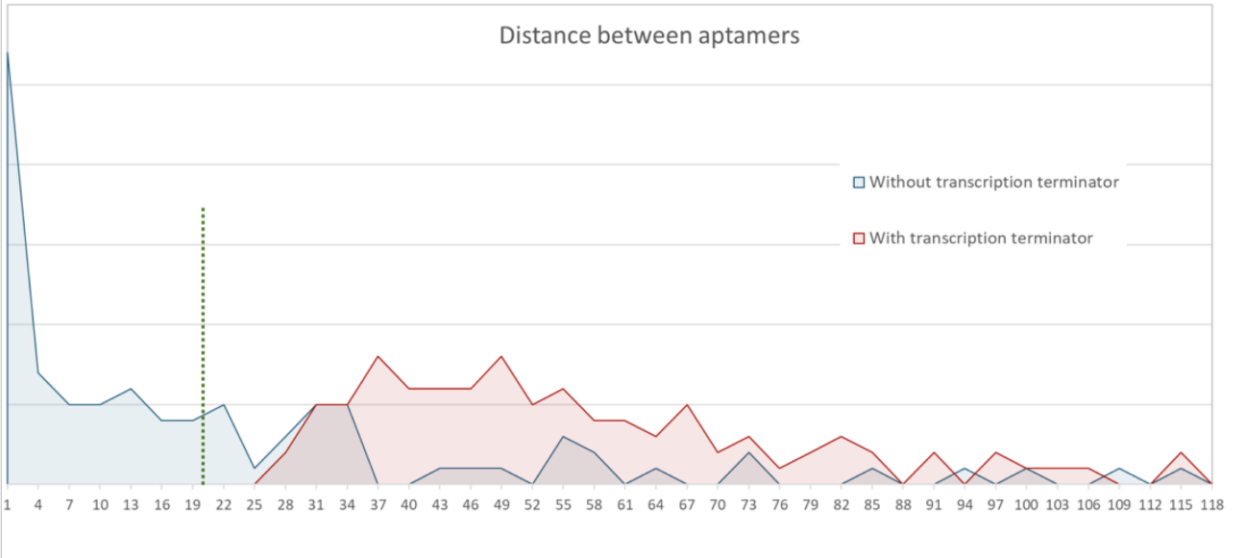

Supplement: Uncited Supplementary Material 1. [file mgen-11-01496-s001.pdf]
